# Shengmai Injection, a Traditional Chinese Patent Medicine, for Intradialytic Hypotension: A Systematic Review and Meta-Analysis

**DOI:** 10.1155/2013/703815

**Published:** 2013-02-07

**Authors:** Chao-yang Chen, Ling-yan Lu, Peng Chen, Kang-ting Ji, Jia-feng Lin, Peng-lin Yang, Ji-fei Tang, Yan Wang

**Affiliations:** Department of Cardiology, The Second Affiliated Hospital of Wenzhou Medical College, Wenzhou, Zhejiang 325027, China

## Abstract

Intradialytic hypotension (IDH) is a global public health problem. A rising number of IDH sufferers resort to Chinese patent medicine, Shengmai Injection (SMI) in China. The objectives of present study are to assess the effectiveness and safety of SMI as an adjunct therapy for IDH. A systematic search of 6 medical databases was performed up to December 2011. Randomized trials involving SMI adjuvant therapy versus conventional therapy were identified. RevMan 5.0 was used for data analysis. Ten randomized clinical trials with 437 participants were identified. Methodological quality was considered inadequate in all trials. Compared with conventional therapy, SMI adjunct therapy showed significant effects in improving the clinic effective rate (*P* < 0.01), decreasing the incidence of IDH episode (*P* < 0.01), decreasing the frequency of nursing interventions (*P* < 0.01), and increasing diastolic blood pressure (*P* < 0.01). There was no statistical significance in the improvement of mean arterial pressure (*P* = 0.22) and systolic blood pressure (*P* = 0.08) between two groups. Four studies had mentioned adverse events, but no serious adverse effects were reported in any of the included trials. In conclusion, SMI adjunct therapy appears to be potentially effective in treatment of IDH and is generally safe. However, further rigorous designed trials are needed.

## 1. Introduction

Intradialytic hypotension (IDH) remains a common and intractable complication for end-stage renal disease (ESRD) patients undergoing hemodialysis [1]. It is defined as a decrease in systolic blood pressure (SBP) by ≥20 mm Hg or a decrease in mean arterial pressure (MAP) by ≥10 mm Hg associated with clinical symptoms (dizziness, blurred vision, cramps, and fatigue), affecting approximately 20% to 30% of dialysis sessions [2, 3]. Frequent hypotension episodes during dialysis not only lead to a discomfortable feeling, limitation of rehabilitation, and consumption of a disproportionate amount of health care resources, but also contribute to high mortality in hemodialysis patients [4, 5]. The etiology leading to IDH is still complex and incompletely understood, but the decline in blood volume, poor cardiac function, and an inadequate cardiovascular response were the main factors [6]. On the basis of the fundamental physiology of blood pressure, the predisposing factors for IDH can be divided into two categories [7]: (1) factors affecting cardiac output such as the decline of cardiac function, blood volume changes during ultrafiltration, and electrolyte changes. The combination of left ventricular hypertrophy, recurrent cardiac ischemic injury, and abnormalities of vascular structure and function may lead to myocardial fibrosis with worsening diastolic function, chamber remodeling, and an increase in electrical excitability and arrhythmias. If the ultrafiltration rate exceeds plasma refilling rates, the plasma volume, preload, and cardiac output will eventually fall. Electrolyte changes can impair myocardial electrical stability and contractility. (2) Factors affecting total peripheral resistance such as autonomic dysfunction (impaired sympathetic response, reduced baroreflex sensitivity, and Bezold-Jarisch reflex), imbalance of vasoactive agents (impaired vasopressin response, elevated adenosine, and increased nitric oxide activity), temperature (thermogenesis and warm dialysate), and immune response to dialysis. Currently, there is no specific consensus on the medical therapy for the prevention and treatment of IDH. Several common therapies were utilized in the past decade including the Trendelenburg position [3], using of cool dialysate, sodium and ultrafiltration profiling, high dialysate calcium, blood volume control, avoidance of food during dialysis, correction of anemia, and pressor agents midodrine [8]. However, it remains necessary to seek novel effective and safe inventions for IDH.

Shengmai San is a well-known traditional Chinese herbal prescription, recorded in *Yixueqiyuan *(*Origins of Medicine*) by Zhang Yuansu at the beginning of 1186 [10], and has been applied for cardiovascular diseases routinely and prophylactically for thousands of years in China [11]. Shengmai San consists of 3 Chinese herbal medicines (CHMs): Renshen (Radix Ginseng; Ginseng), Maidong (Radix Ophiopogonis; Dwarf Lilyturf Tuber), and Wuweizi (Fructus Schisandrae Chinensis; Chinese Magnoliavine Fruit). All three herbs of SMI are included in the Chinese Pharmacopoeia (version 2010). Theory of traditional Chinese medicine believes that Shengmai San has the effect of supplementing Qi and nourishing Yin, recovering pulse, and stopping abnormal sweating. Shengmai injection (SMI), which is developed on the basis of Shengmai San, is a popular modern Chinese patent herbal preparation. SMI is widely used in various cardiovascular diseases, and at least three systematic reviews to date have been conducted to evaluate the effectiveness of SMI on heart failure [12], fatality rate of acute myocardial infarction [13], and hypotension after acute myocardial infarction [14]. 

Evidences have accumulated from former experiments to confirm the effect of SMI on regulating blood pressure [15]. Especially, the widespread use of SMI on hypotension due to a variety of causes is noteworthy [14]. SMI can significantly elevate blood pressure in hypotensive patients no matter if it is essential hypotension or with secondary reasons [14, 16, 17]. However, SMI has no significant effect on blood pressure in healthy subjects [18]. 

Pharmacological studies have revealed the effects of SMI on multiaspects of the pathophysiology of IDH [7]. The related pharmacological mechanisms of SMI were as follows: (1) SMI can improve cardiac function through the protection of myocardial cells, reduction of ischemia-reperfusion injury, reduction of myocardial apoptosis, prevention of myocardial calcium overload and alleviation of myocardial hypertrophy, enhancement of myocardial contractility, and protection of endothelial function [19]; (2) SMI can inhibit local angiotensin II activity so as to alleviate left ventricular hypertrophy [20]; (3) SMI had protective effects against oxidative damage in mitochondria, cells, and tissues [21, 22]; (4) SMI had protective effects against experimental acute cardiogenic shock by improving the hemodynamics parameter [23]; (5) SMI can inhibit high sensitive C-reactive protein (hs-CRP) and inflammatory cytokines such as tumor necrosis factor-*α* and interleukin-8 and reduce the systemic inflammatory reaction [24, 25]; (6) SMI can enhance humoral immunity and inhibit the cellular immunity after cardiopulmonary bypass [26]; (7) SMI can increase sympathetic tone, enhance sinus node function, and improve conduction [27]. (8) Impressively, Shengmai San can significantly attenuate heat stroke-induced arterial hypotension and cerebral ischemia through inhibition of inducible nitric oxide synthase-(iNOS-) dependent nitric oxide (NO) overproduction in the brain and excessive accumulation of inflammatory cytokines like interleukin-1 beta, interleukin-6, and tumor necrosis factor-alpha in the peripheral blood stream [28]. In addition, Ginseng, as the principal drug in the SMI, showed the effect of improving blood pressure stability in IDH patients. Chewing Korean red ginseng could significantly reduce the degree of blood pressure drop during hemodialysis and the frequency of symptomatic IDH, and this beneficial effects may be partially due to decreased NO production and more activation of vasoconstrictors including endothelin-1, renin activity (PRA), and angiotensin II (Ang II) [29].

However, the exact active ingredients of SMI for IDH treatment are still unclear. For the chemical composition of the individual Chinese herb of SMI, ginsenoside, ophiopogonin and ophiopogonone, and lignan have been proposed as the active components of Radix Ginseng, Radix Ophiopogonis, and Fructus Schisandrae Chinensis, respectively [30]. There are a number of reports about the effective chemical constitutes and different analytical methods for analyzing constituents in SMI. High performance liquid chromatography (HPLC) have even been widely employed for content determination of Shengmai preparations [31]. Recently, by the use of the liquid chromatography-electrospray ionization source in combination with hybrid ion trap and high-resolution time-off light mass spectrometry (LC-IT-TOF/MS), more than 30 ginsenosides and 20 lignans were readily detected and structurally characterized from SMI [30]. Interestingly, by using the on-line high performance liquid chromatography-diode array detection-chemiluminescence (HPLC-DAD-CL) method and liquid chromatography coupled with tandem mass spectrometry (LC/MS/MS) analysis, the scavenging activities of main components detected in the individual herb were different from those in whole Shengmai San, suggesting that drug interactions in complex multiherbal formula could change the activity of the constituents [32]. 

Over the past decades, a number of trials have indicated that SMI could have therapeutic potential in people with IDH in China. However, the evidences for the effects of SMI have not been systematically assessed. The objective of the present study is thus to assess the clinical effectiveness and safety of SMI adjunct therapy for IDH patients.

## 2. Methods

This systematic review is conducted according to the paper [9].

### 2.1. Eligibility Criteria

#### 2.1.1. Types of Studies

Only the randomized controlled clinical trials (RCTs) that evaluate the effects of SMI as adjunct therapy for IDH patients were included, regardless of blinding, publication status, and language. Quasi-RCTs were not considered such as using the admission sequence for treatment allocation.

#### 2.1.2. Types of Participants

Patients of any age or sex with end-stage renal disease (ESRD) who were receiving long-term regular hemodialysis and had experienced episodes of IDH were included. The diagnostic criteria were adopted in accordance with the following. (1) Diagnosis of IDH was made on the basis of “Definition of IDH” in 2005 from the European Dialysis and Transplant Association and K/DOQI guideline, a decrease in SBP ≥20 mm Hg or a decrease in mean arterial blood pressure (MAP) ≥10 mm Hg associated with dialysis-related hypotension symptoms [2]. (2) Diagnostic criteria of IDH with comparable definitions was made on the basis of *blood purification*, second edition written by Wang in 2003, a reduction in SBP below 90 mm Hg, or a decrease in SBP ≥20 mm Hg from prehemodialysis [33]. None of them received antihypertensive drugs or any other intervention known to influence the blood pressure before dialysis.

#### 2.1.3. Types of Interventions

SMI in any dose compared with the conventional therapy for IDH was considered. We only included studies that compared SMI with conventional therapy. Studies comparing SMI with another CHM were excluded.

### 2.2. Outcome Measures

The outcome measures included the clinical effective rate of SMI for IDH, the incidence rate of hypotension, the number of nursing interventions, blood pressure level, and adverse events. Clinical effectiveness is defined as the ability of SMI to improve hemodynamics and clinical symptoms related to IDH. Evaluation standards for clinical therapeutic effects were as follows [34]: (1) markedly effective: the SBP increased more than 20 mm Hg or SBP >90 mm Hg or MAP increased by ≥10 mm Hg compared with pretreatment, with no hypotension-related symptoms, and dialysis tobe completed successfully; (2) effective: SBP increased by 10~20 mm Hg or SBP >90 mm Hg or MAP increased by ≥0–10 mm Hg compared with pretreatment, with no obvious symptoms of low blood pressure, and dialysis to be completed by adjusting the dialysis program; (3) ineffective or deterioration: blood pressure did not rise or continued to decline, SBP dropped to less than 90 mm Hg, and patients showed significant symptoms of low blood pressure, need vasopressors, volume expansion and other drug treatment to maintain blood pressure or were forced to interrupt dialysis.

### 2.3. Search Strategy

We conducted electronic searches in the following databases: Cochrane Central Register of Controlled Trials (2011, issue1), Pubmed (December 1950–2011), EMBASE (1980–2011), Chinese Hospital Knowledge Database (CHKD, December 1979–2011), Wanfang Med Online Database (WMOD, December 1998–2011). We also checked the references of published studies to identify additional trials.

The following search terms were used as medical subject headings and key words when searching electronic databases: end-stage renal disease, end-stage renal failure, end-stage kidney failure, Shengmai, Sheng-mai Injection, hemodialysis related hypotension, intradialytic hypotension, IDH, and low blood pressure. These terms were used as Mesh and free-text terms (translated into Chinese) to search the Chinese databases.

### 2.4. Study Selection and Data Extraction

Two review authors (C.-y. Chen, L.-y. Lu) independently examined the titles and abstracts of the potential references. Full articles for all potentially relevant studies were retrieved. The two reviewers then read the selected papers independently and made a final selection decision. Disagreements were resolved through discussion or consultation with a third author (Y. Wang). If necessary. The authors of the trials were contacted and asked to provide missing data.

The review authors extracted data on study characteristics, including patients, methods, interventions, and outcomes, into a standardized data extraction form. Reasons for the exclusion of studies were recorded. For eligible studies, two review authors (C.-y. Chen, L.-y. Lu) extracted data independently. Any disagreements were resolved by consensus or by a third reviewer (Y. Wang).

### 2.5. Risk of Bias in Individual Studies

Assessment of risk of bias in included studies: two review authors (C.-y. Chen, L.-y. Lu) independently assessed risk of bias for each included article, using the twelve criteria recommended by the Cochrane Back Review Group [35]. The items were scored with “yes (+),” “no (−),” or “unsure (?).” Disagreements were resolved through discussion with or involving a third author (Y. Wang).

### 2.6. Data Synthesis and Analysis

The statistical package RevMan 5.0 provided by the Cochrane Collaboration was used to analyze the data. Dichotomous data were presented as odds ratio (OR), with 95% confidence intervals (CI). Continuous outcomes were presented as weighted mean difference, with 95% CI. Meta-analysis was only performed within comparisons where individual trials compared similar treatment and control interventions.

## 3. Results

### 3.1. Description of Studies

**Figure 1:**
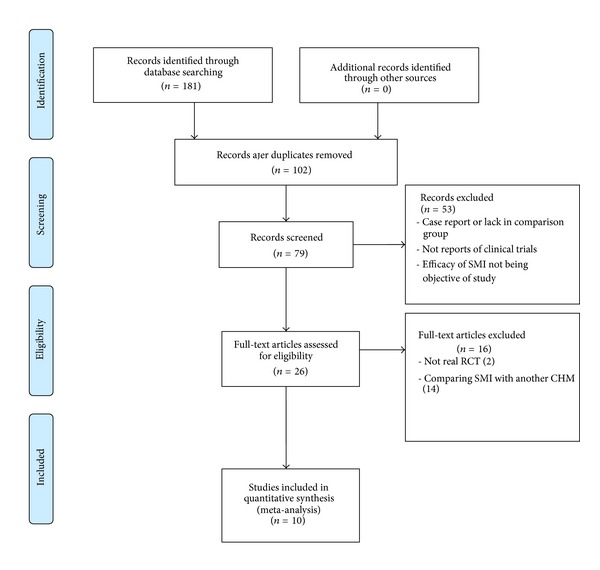
PRISMA 2009 flow diagram, from [9]. For more information, visit http://www.prisma-statement.org/statement.htm.

We identified and screened 181 potentially relevant articles. Of these, 102 articles were initially excluded due to duplicate publications by reading titles and abstracts, and 53 articles were excluded because they were case reports or lack in-comparison group, or not reports of clinical trials, or effectiveness of SMI not being objective of the studies. In the identified 26 potentially eligible reports, after reading the full text, 14 articles were excluded due to comparing SMI with another CHM, and 2 more articles were excluded because they were not real RCTs with hemodialysis order used for treatment allocation [36, 37]. Therefore, a total of 10 studies were finally included papers [38–47]. Flow diagram was summarized in Figure 1.

### 3.2. Characteristics of Included Studies

A total of 437 participants were involved in the 10 studies included (Table 1). All studies were conducted in China and published between 1999 and 2010 on Chinese journals. Each study was performed in a single center, parallel-designed, and claimed to have applied randomization. 8 studies included 180 male and 124 female, while the other 2 studies did not mention the gender condition [44, 46]. The age of the participants ranged from 15 years to 78 years. Etiology for ESRD was introduced in 184 patients in 5 studies [38, 41–43, 47], including 107 chronic glomerulonephritis, 15 diabetic nephropathy, 35 hypertensive nephropathy, 10 obstructive nephropathy, 2 polycystic renal disease, 1 chronic pyelonephritis, 5 gouty nephropathy, and 9 other types of nephropathy. 3 studies reported the modality of dialysis, on bicarbonate dialysis for 4-5 hrs and 2-3 times a week with a low-flux polysulfone hollow-fiber dialyzer [40, 41, 45]. 6 studies reported the duration of the dialysis from one month to 5 years [38, 39, 41–43, 45]. All of the 10 included trials were two-group parallel design studies. 

In the interventions, conventional therapy referred to treatment according to the European Dialysis and Transplant Association and K/DOQI guidelines, including the use of cool dialysate, sodium and ultrafiltration profiling, high dialysate calcium, blood volume control, avoidance of food during dialysis, correction of anemia, and the use of pressor agents such as midodrine [2, 8]. The doses of SMI used ranged from 40 mL to 60 mL. SMI was administered intravenously in all included studies. A variety of outcome measures were reported. Evaluation of the outcomes was performed at the end of the treatment.

### 3.3. Risk of Bias in Included Studies

The risk of bias of each study was assessed using the twelve criteria recommended by Cochrane Back Review Group. The number of criteria met varied from 2/12 to 5/12. All of the studies included claimed randomization. No study described allocation concealment. No trials mentioned the blinding procedures. One study described intention-to-treat analyses [47]. None of the trials mentioned drop-out data. There was selective reporting in all the studies. All the studies described similarity of baseline except two studies [38, 40]. All of the included studies appeared to have adequate and acceptable compliance and timing of outcome assessments were similar. In general, all of 10 RCTs have an unclear risk of bias. The methodological quality of each study is summarized in Table 2.

### 3.4. Results of Individual Studies

Zhao et al. [38] conducted an RCT to test the effect of SMI on correcting IDH. 100 hemodialysis sessions were divided into two subgroups: the treatment group received SMI 40 mL intravenously, and the control group received normal saline injection. The results showed that the total clinical effective rate was 85% in treatment group and 55% in control group (*P* < 0.01). 

In the study of Liu and Su [39], 70 IDH patients were randomly divided into experimental group and control group. The experimental group received SMI 60 mL intravenously plus conventional therapy, while only conventional therapy was given for control group. The total clinical effective rate was 88.57% in experimental group and 62.86% in control group (*P* < 0.05). The frequency of fluid infusion treatment in experimental group was significantly lower than that in the control group (*P* < 0.05).

Zhou [40] recruited 14 patients (totally 140 hemodialysis sessions) and randomly divided into two groups. The therapy group was given SMI 60 mL intravenously and the control group was given 50% glucose 60 mL correspondingly. The result showed that the hypotension rate was 8% in therapy group and 38% in control group (*P* < 0.01).

In the study of Zheng et al. [41], patients in treatment group were additionally given SMI 50 mL intravenously. The hypotension rate was 18.8% in treatment group and 33.1% in control group (*P* < 0.01). SBP, diastolic blood pressure (DBP), and MAP were all significantly higher in treatment group than in control group (*P* < 0.01). The difference of MAP between the two groups was also statistically significant (*P* < 0.05).

Jiang et al. [42] selected 18 patients (352 hemodialysis sessions) and randomly divided them into two groups. Patients in control group were given midodrine hydrochloride tablet before and after dialysis. Patients in therapy group were additionally given SMI 60 mL intravenously on that basis. The overall effective rate was 88.5% in therapy group and 69.7% in control group (*P* < 0.05). SBP, DBP, and MAP were all significantly higher in therapy group compared with control group (*P* < 0.01). The difference of MAP after dialysis was significant between the two groups (*P* < 0.05).

In the study of Lv and Liu [43], patients of treatment group received SMI 60 mL treatment, while patients of control group only received 0.9% saline. There were no significant differences between two groups in MAP, systolic pressure, diastolic pressure, and heart rate (*P* > 0.05). Clinical effective rate in experimental group was significantly higher than control group (*P* < 0.05). Number of measures taken to rectify the dialysis-related symptoms were treatment group 2.3 ± 1.2 times and control group 5.4 ± 1.5 times. The difference was statistically significant (*P* < 0.05).

In the study of Wang [44], the total effectiveness rate was 86.8% in SMI group and 62.9% in conventional therapy group (*P* < 0.05). Number of measures taken to rectify the dialysis-related symptoms were: SMI group 2.3 ± 1.5 times, conventional therapy group 5.1 ± 1.3 times (*P* < 0.05).

In the study of Yu [45], control group was given 50% glucose + conventional therapy. Treatment group was given SMI 60 mL + conventional therapy. The rate of hypotension in treatment group was significantly lower than that of control group (*P* < 0.01). The clinical effective rate in treatment group was higher than that of control group (*P* < 0.05). 

Cao et al. [46] recruited 60 cases of IDH patients and randomly divided them into 2 groups: SMI group and conventional group. The mean arterial blood pressure of SMI group was, predialysis: 96.4 ± 13.1 mm Hg; postdialysis: 97.8 ± 9.1 mm Hg; conventional group, predialysis: 99.2 ± 9.5 mm Hg; postdialysis: 99.7 ± 8.6 mm Hg. Number of measures taken to rectify the dialysis-related symptoms were SMI group: 2.4 ± 1.1 times; conventional group: 5.4 ± 1.8 times. There was a significant difference between the two groups (*P* < 0.05).

In the study of Li [47], the therapy group was given the following treatment: SMI + 50% glucose, i.v., while the control group was given 0.9% sodium chloride injection or 20% human albumin or fresh plasma, ivgtt. Results showed that level of blood pressure and improvement of clinical symptoms were significantly better in therapy group than in control group (*P* < 0.01).

### 3.5. Synthesis of Results

#### 3.5.1. The Clinical Effective Rate

**Figure 2:**
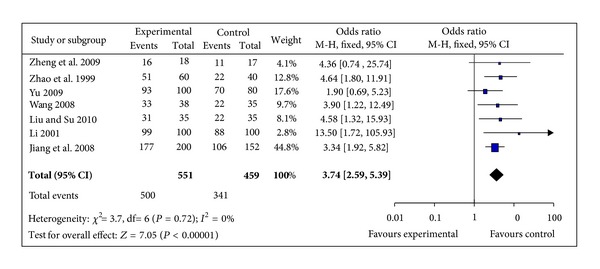
Forest plot of comparison: shengmai injection versus control, the clinical effective rate.

**Figure 3:**
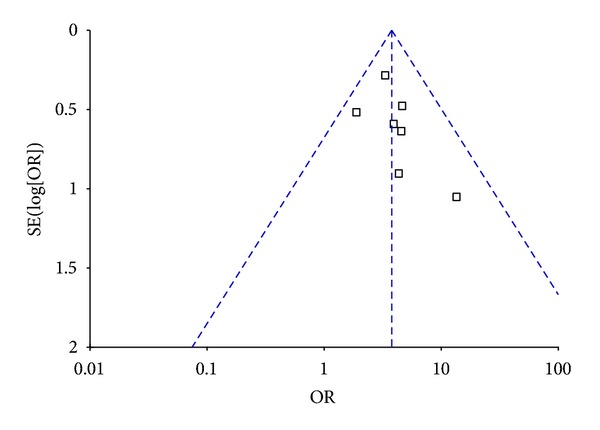
Funnel plot of comparison: shengmai injection versus control.

7 trials [38–41, 44, 45, 47] calculated the clinical effective rate with the ratio between the proportion of responders in the treatment group and in the control group. The 7 independent trials showed homogeneity in the consistency of the trial results (chi-square = 3.70, *P* = 0.72, *I*² = 0%). Thus, fixed-effects model should be used for statistical analysis. The combined effects showed that patient with IDH receiving SMI therapy had significantly improved the clinical effective rate when compared with the control group (OR 3.74, 95% CI 2.59 to 5.39; *Z* = 7.05,  *P* < 0.00001), Figure 2. The funnel plot was roughly symmetric. There would be little publication bias for the 7 independent trials (Figure 3).

#### 3.5.2. The Incidence of Hypotension

**Figure 4:**
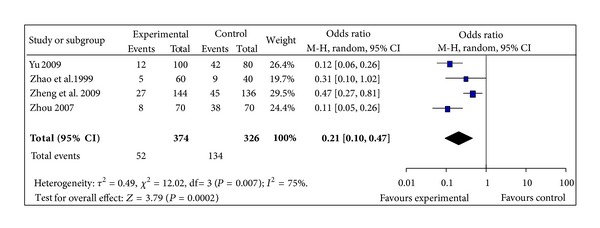
Forest plot of comparison: sheng-mai injection versus control: Hypotension incidence.

4 studies observed the incidence of IDH episode [38, 40, 41, 45]. The 4 trials did not show homogeneity (chi-square 12.02, *P* = 0.007, *I*² = 75%). Thus, random effects model should be used for statistical analysis. SMI treatment could significantly decrease the incidence of IDH episode (OR 0.21, 95% CI 0.10 to 0.47, *Z* = 3.79,  *P* = 0.0002), Figure 4.

#### 3.5.3. The Number of Nursing Interventions

**Figure 5:**
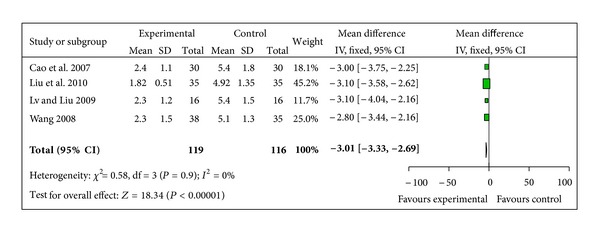
Forest plot of comparison: sheng-mai injection versus control: The number of nursing interventions.

4 studies recorded the number of nursing interventions for IDH episode [39, 43, 44, 46]. Routine nursing interventions are as follows: placing the patient in the Trendelenburg position, saline and hyperoncotic albumin boluses, decreasing the transmembrane ultrafiltration pressure, and early termination of dialysis. The 4 trials showed homogeneity in the results (chi-square = 0.58,  *P* = 0.90, *I*² = 0%). Thus, fixed effects model should be used for statistical analysis. There was a significant decrease on frequency of nursing interventions in SMI group (WMD −3.01, 95% CI −3.33 to −2.69,  *Z* = 18.34, *P* < 0.00001), Figure 5.

#### 3.5.4. Blood Pressure Level

**Figure 6:**
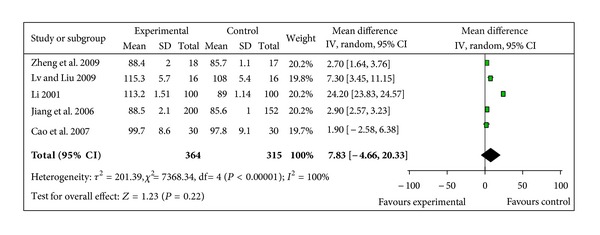
Forest plot of comparison. Shengmai injection versus control: mean arterial pressure.

**Figure 7:**
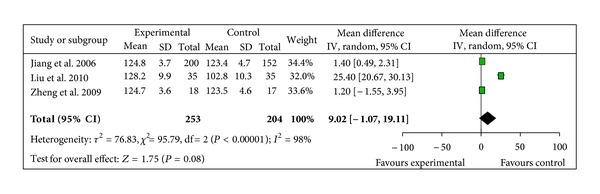
Forest plot of comparison. Shengmai injection versus control: systolic blood pressure.

**Figure 8:**
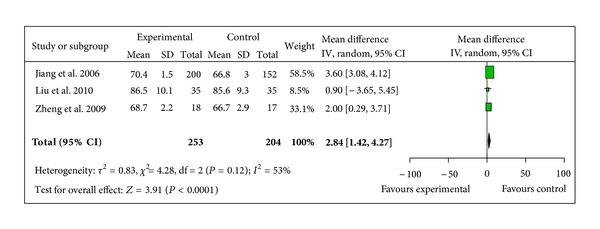
Forest plot of comparison. Shengmai injection versus control: diastolic blood pressure.

BP change was reported in 3 different ways across the studies: pre- and post-SBP, pre- and post-DBP, and pre- and post-MAP. 5 trials provided data for pre- and post-MAP change [41–43, 46, 47]. The 5 trials did not show homogeneity in the trial results (chi-square 7368.34, *P* < 0.00001, *I*² = 100%). Thus, random-effects model should be used for statistical analysis. There was no statistical significance in increasing MAP between two groups (WMD 7.83, 95% CI −4.66 to 20.33, *Z* = 1.23, *P* = 0.22), Figure 6. 3 studies reported pre- and post-SBP, and pre- and post-DBP [39, 41, 42]. The trials did not show homogeneity in the trial results, thus random-effects model should be used for statistical analysis. There was no statistical significance in increasing SBP when compared with control group (WMD 9.02, 95% CI −1.07 to 19.11, *Z* = 1.75, *P* = 0.08), Figure 7, but there was a significant increase in DBP in SMI group (WMD 2.84, 95% CI 1.42 to 4.27, *Z* = 3.91, *P* < 0.0001), Figure 8.

#### 3.5.5. Adverse Events

Four studies reported nonserious adverse events [38, 41, 42, 47]. The other 6 studies did not report adverse events [39, 40, 43–46]. Zhao et al. [38] indicated no statistically significant difference in serum creatinine, blood urea nitrogen, serum electrolytes, and electrocardiogram before and after hemodialysis in treatment group and control group (*P* > 0.05). There was no case report of toxic side effects or allergy in treatment group. Zheng et al. [41] found no significant change in heart rate before and after dialysis. There were no adverse reactions in the two groups during dialysis. Jiang et al. [42] reported that no significant difference in heart rate before and after dialysis in the two groups. There was no significant difference in routine blood test, blood urea nitrogen, creatinine, alanine aminotransferase, albumin, urea clearance index (*Kt/V*) in the two groups before and after treatment (*P* > 0.05). There were no adverse reactions in patients of the two groups, and the treatment was well tolerated. Li [47] demonstrated that the side effects in SMI group were lower than that of control group. In the control group, allergic reactions and transfusion reactions occurred in 4 cases, heart failure in 2 cases, dialyzer clotting in 8 cases, and early termination of dialysis was 6 cases due to no improvement of clinical symptoms and blood pressure. In the SMI group, dialyzer clotting occurred in 1 case and could continue hemodialysis after replacing the dialyzer. All patients completed the expected dialysis and no adverse reactions such as allergic reactions, abdominal distension, tachycardia, and hypotension happened.

## 4. Discussion

### 4.1. Summary of Evidence

10 studies with 437 individuals suffering from IDH were selected out for the present meta-analysis. The main findings are that SMI adjuvant therapy could improve the clinical symptoms of IDH, decrease the incidence of hypotension, reduce the number of nursing intervention, increase DBP, and reduce the adverse effects. However, the evidences presented in this meta-analysis are insufficient to warrant a clinical recommendation due to the generally weak methodological quality of the included studies.

### 4.2. Limitations

Weaknesses of this paper rest with inherent limitations in the primary studies. In September 2004, the members of the International Committee of Medical Journal Editors (ICMJE) published a statement requiring that all clinical trials must be registered in order to be considered for publication [48]. However, none of the included studies in this paper had been formally registered in WHO International Clinical Trials Registry Platform. Thus, protocols were not available to confirm free of selective reporting.

There are also a number of methodological limitations in this meta-analysis. Firstly, the data were all collected from the published articles without directly contacting the authors for obtaining additional information about the included studies. Therefore, the twelve criteria of the “risk of bias” assessment tool could only be classified as “unclear.” Secondly, all studies included in this paper used an “A+B versus B” design where patients were randomized to receive SMI plus conventional therapy versus conventional therapy, without a rigorous control for placebo effect. This kind of design is likely to generate false positive results [49]. Thirdly, all 10 studies claimed to be RCTs, but they all failed to give adequate and convincing information on how the random allocation was generated and concealed, which is necessary to avoid selection bias. They also did not mention blinding method, and thus could produce performance bias and detection bias. Therefore, outcome assessment was prone to significant systemic errors. Intention-to-treat analysis was mentioned only in one study [47], and no dropouts were reported. Thus, the results generated from these studies should be interpreted with caution. Fourthly, the included studies were of relatively small sample size and without formal sample size calculation. Trials that lacked proper sample size estimation placed their statistical analysis's validity in doubt. Baseline information on ESRD patients was insufficient, with 6 trials provided information on chronic hemodialysis duration [38, 39, 41–43, 45] and 5 studies reported the etiology of ESRD [38, 41–43, 47]. Varying dialyser, dialysis, membrane and dialysate were used in different studies. The lack of baseline information may lead to selection bias and not to comparable baseline.

No study found severe adverse effects of SMI. Due to the small sample size, safety still needs to be assessed. Publication bias may also exist because only Chinese language publications were found and included.

## 5. Conclusions

### 5.1. Implications for Practice

This is the first meta-analysis of randomized, controlled trials to assess the effectiveness and safety of SMI adjuvant therapy in patients with IDH. However, the evidences available from this systematic review is insufficient to recommend the routine use of SMI as adjuvant therapy for IDH, because the strength of the evidences is compromised by methodological flaws and lack of replicable validation. The effectiveness and safety of SMI therapy for IDH remain to be further determined.

### 5.2. Implications for Research

First, improvement in the methodological quality of randomized controlled trials is critical for future research and more methodologically rigorous studies are justified to confirm or refute the effects reported here. Second, the included trials were generally of small sample size. All the trials were in lack of sample size estimation, so sample size calculation should be conducted before enrollment. Relevant clinical events such as death, dependency, and activities of daily living at the longer followup period should be included in outcome assessment. Third, well-designed, randomized, double-blind, placebo-controlled trials need to be carried out and reported in detail according to CONSORT [50] or CONSORT for TCM [51, 52].

## Figures and Tables

**Table 1:** Characteristics of included studies.

Include studies		Study Designs	Participants				Duration of dialysis	Cause of renal failure								Dialysis equipment	Modality of dialysis	Interventions		Outcomes
Date of study	First author		*n*	T/C	Age (mean or rang T/C) yrs	Gender M/F		CGN	CPN	ON	PRD	GN	DIN	HTN	O	Dialyser Dialysis membrane Dialysate		Experiment group	Control group	
1999	Zhao	RCT	49	60/40*	21–65	28/21	0–5 yrs	40					2	3	4	Not mentioned	Not mentioned	SMI 40 mL + 5% glucose solution 200 mL ivgtt. 90–100 drops/min	0.9% Sodium Chloride solution 240 mL ivgtt. 90–100 drops/min	Clinical effect Scr Bun IDEM ECG Clinical symptom Adverse effect

2010	Liu	RCT	70	35/35	(53.96 ± 13.23)	42/28	6 months –5 yrs	Not mentioned								Not mentioned	Not mentioned	SMI 60 mL + 0.9% sodium chloride solution 250 mL ivgtt. + conventional therapy	Conventional therapy (change dialysate temperature, reduce or stop ultrafiltration, slow down blood flow, increase sodium concentration in dialysate, 50% glucose solution 250 mL ivgtt rapidly)	Clinical effect SBP DBP HR

2007	Zhou	RCT	14	70/70*	15–78 (42)	8/6	Not mentioned	Not mentioned								GambroAK90S Polysulfon Bicarbonate	4 h* 2-3/Week	SMI 60 mL + 0.9% sodium chloride solution 250 mL ivgtt.	50% glucose solution 60 mL + 0.9% sodium chloride solution 250 mL ivgtt.	Hypotension incidence

2009	Zheng	RCT	35	18/17 144/136*	(61.25)	22/13	>1 yrs	18	1	6				8	2	Fresenius4008S Polysulfon Bicarbonate	4 h* 3/Week	Shengmai injection 50 mL + 50% glucose 100 mL ivgtt. continuously during dialysis	50% glucose 100 mL ivgtt. continuously during dialysis	Hypotension incidence BP HR Clinical effect Adverse effect

2006	Jiang	RCT	18	200/152*	43–78 (61.2 ± 12.3)	10/8	33.5 ± 7.6 mo	10		2	2	1	3			Not mentioned	Not mentioned	30 min before dialysis: midodrine hydrochloride Tablet 5 mg po.; 1 h after dialysis: 5 mg po.; + SMI 60 mL ivgtt + conventional measures Once a day, 15 days for a course of treatment, lasting for 2-3 courses.	30 min before dialysis: midodrine hydrochloride Tablet 5 mg po.; 1 h after dialysis: 5 mg po. + conventional therapy (infusion of hypertonic liquid, or reduce the amount of ultrafiltration, or even stop dialysis.) Once a day, 15 days for a course of treatment, lasting for 2-3 courses.)	Clinical effect SBP DBP MAP HR Adverse effect

2009	Lv	RCT	32	16/16	(66.3)	18/14	>3 mo	9					10	12	1	GambroAK200 Cellulose acetate Bicarbonate	Not mentioned	SMI 60 mL + 0.9% sodium chloride solution 40 mL ivgtt.	0.9% Sodium Chloride solution 100 mL ivgtt.	MAP The number of nursing interventions.

2008	Wang	RCT	73	38/35	T: 60–72 C: 60–71	Not mentioned	Not mentioned	Not mentioned								Fresenius 4008B Cellulose acetate Bicarbonate	Not mentioned	SMI: no detailed information was provided.	Conventional therapy: no detailed information was provided.	Clinical effect The number of nursing interventions.

2009	Yu	RCT	36	100/80*	53.5	20/16	3 mo–3.5 yrs	Not mentioned								Fresenius 4008H/S Polysulfon Bicarbonate	4 h* 2-3/Week	SMI 60 mL + 0.9% sodium chloride solution 250 mL ivgtt. + conventional therapy	50% glucose solution 250 mL ivgtt + conventional therapy (reduce or stop ultrafiltration, slow down blood flow, increase sodium concentration in dialysate)	Hypotension incidence Clinical effect

2007	Cao	RCT	60	30/30	T: (62.1 ± 14.4) C: (60.0 ± 14.0)	Not mentioned	Not mentioned	Not mentioned								GambroAK200 Cellulose acetate Bicarbonate	Not mentioned	SMI: no detailed information was provided.	Conventional therapy: no detailed information was provided.	MAP The number of nursing interventions.

2001	Li	RCT	50	100/100*	18–75 (48.8)	32/18	Not mentioned	30		2		4		12	2	Gambro AK-10, AK-90, AK-200 Bicarbonate	Not mentioned	SMI 10–40 mL + 50% glucose solution 20–40 mL iv..	0.9% saline 300~500 mL, or 20% human albumin 50 mL, or fresh plasma 200~400 mL ivgtt.	MAP Clinical effect Adverse effect

RCT: randomized controlled trial; HD: hemodialysis; T/C: treatment group/control group; M/F: male/female; yrs: years; CGN: chronic glomerulonephritis; CPN: chronic pyelonephritis; ON: obstructive nephropathy; PRD: polycystic renal disease; GN: gouty nephropathy; DIN: diabetic nephropathy; HTN: hypertensive nephropathy; O: other; SMI: shengmai injection; SBP: systolic blood pressure; DBP: diastolic blood pressure; HR: heart rate; MAP: mean arterial pressure. *hemodialysis sessions.

**Table 2:** The methodological quality of included studies.

	A	B	C	D	E	F	G	H	I	J	K	L	Total +/12	Total −/12	Total ?/12
Zhao et al. 1999 [38]	?	?	−	−	?	−	−	?	?	+	+	+	3	4	5
Liu and Su 2010 [39]	?	?	−	−	?	−	−	?	+	+	+	+	4	4	4
Zhou 2007 [40]	?	?	−	−	?	−	−	?	?	+	+	+	3	5	5
Zheng et al. 2009 [41]	?	?	−	−	?	−	−	?	+	+	+	+	4	4	4
Jiang et al. 2006 [42]	?	?	−	−	?	−	−	?	+	+	+	+	4	4	4
Lv and Liu 2009 [43]	?	?	−	−	?	−	−	?	+	+	+	+	4	4	4
Wang 2008 [44]	?	?	−	−	?	−	−	?	+	+	+	+	4	4	4
Yu 2009 [45]	?	?	−	−	?	−	−	?	+	+	+	+	4	4	4
Cao et al. 2007 [46]	?	?	−	−	?	−	−	?	+	+	+	+	4	4	4
Li 2001 [47]	?	?	−	−	?	+	−	?	+	+	+	+	5	3	4

A: adequate sequence generation; B: concealment of allocation; C: blinding (patient); D: blinding (investigator); E: blinding (assessor); F: incomplete outcome data addressed (ITT analysis); G: incomplete outcome data addressed (dropouts); H: free of selective reporting; I: similarity at baseline; J: cointerventions constant; K: Compliance acceptable; L: timing outcome assessments similar. +: Yes, −: No, ?: unclear.
